# 3D interconnected periodic carbon-tube membrane enabled self-cleaning solar brine treatment and autonomous salt production

**DOI:** 10.1093/nsr/nwag188

**Published:** 2026-04-06

**Authors:** Ning Xu, Qijun Pan, Chun Shen, Ou Qian, Shiqi Fang, Fangming Han, Mengyue Zeng, Songguo Li, Jia Zhu, Wanlin Guo, Guowen Meng

**Affiliations:** National Laboratory of Solid State Microstructures, College of Engineering and Applied Sciences, Frontiers Science Center for Critical Earth Material Cycling, Jiangsu Key Laboratory of Artificial Functional Materials and Collaborative Innovation Center of Advanced Microstructures, Nanjing University, Nanjing 210093, China; Key Laboratory of Materials Physics and Anhui Key Laboratory of Nanomaterials and Nanotechnology, Institute of Solid State Physics, HFIPS, Chinese Academy of Sciences, Hefei 230031, China; University of Science and Technology of China, Hefei 230026, China; State Key Laboratory of Mechanics and Control for Mechanical Structures, Nanjing University of Aeronautics and Astronautics, Nanjing 210016, China; Key Laboratory of Materials Physics and Anhui Key Laboratory of Nanomaterials and Nanotechnology, Institute of Solid State Physics, HFIPS, Chinese Academy of Sciences, Hefei 230031, China; University of Science and Technology of China, Hefei 230026, China; National Laboratory of Solid State Microstructures, College of Engineering and Applied Sciences, Frontiers Science Center for Critical Earth Material Cycling, Jiangsu Key Laboratory of Artificial Functional Materials and Collaborative Innovation Center of Advanced Microstructures, Nanjing University, Nanjing 210093, China; Key Laboratory of Materials Physics and Anhui Key Laboratory of Nanomaterials and Nanotechnology, Institute of Solid State Physics, HFIPS, Chinese Academy of Sciences, Hefei 230031, China; University of Science and Technology of China, Hefei 230026, China; National Laboratory of Solid State Microstructures, College of Engineering and Applied Sciences, Frontiers Science Center for Critical Earth Material Cycling, Jiangsu Key Laboratory of Artificial Functional Materials and Collaborative Innovation Center of Advanced Microstructures, Nanjing University, Nanjing 210093, China; National Laboratory of Solid State Microstructures, College of Engineering and Applied Sciences, Frontiers Science Center for Critical Earth Material Cycling, Jiangsu Key Laboratory of Artificial Functional Materials and Collaborative Innovation Center of Advanced Microstructures, Nanjing University, Nanjing 210093, China; National Laboratory of Solid State Microstructures, College of Engineering and Applied Sciences, Frontiers Science Center for Critical Earth Material Cycling, Jiangsu Key Laboratory of Artificial Functional Materials and Collaborative Innovation Center of Advanced Microstructures, Nanjing University, Nanjing 210093, China; School of Sustainable Energy and Resources, Nanjing University, Suzhou 215163, China; State Key Laboratory of Mechanics and Control for Mechanical Structures, Nanjing University of Aeronautics and Astronautics, Nanjing 210016, China; Key Laboratory of Materials Physics and Anhui Key Laboratory of Nanomaterials and Nanotechnology, Institute of Solid State Physics, HFIPS, Chinese Academy of Sciences, Hefei 230031, China; University of Science and Technology of China, Hefei 230026, China

**Keywords:** three-dimensional carbon-tube membrane, solar brine treatment, autonomous self-cleaning, salt production

## Abstract

Interfacial solar vapor generation, an emerging solar technology, shows great potential for efficient and sustainable water treatment. With the solar thermal energy localized in the interfacial water layer, this approach significantly enhances the evaporation rate. However, as the interfacial water layer typically exists in the evaporator, precipitated salts tend to adhere to the evaporator during evaporation. This not only brings unavoidable performance degradation due to the blockage of incident sunlight and vapor-escaping channels, but also causes a waste of salt resources. Herein, we propose a concept of an interfacial water layer on the evaporator (IWOE), featuring self-cleaning solar brine treatment and autonomous salt production. Further, we experimentally demonstrate this concept by using a rationally tailored 3D carbon-tube (3D-CT) membrane, which reaches an elegant equilibrium of gravity, buoyancy, and surface tension under water. During the solar treatment of saturated brine, salt crystals precipitate and grow within this interfacial water layer, leading to autonomous tilt of the membrane and glide of the salt crystals. Thus, the 3D-CT membrane with IWOE enables autonomous self-cleaning solar brine treatment and salt production from high-concentration brine, showing potential applications in various fields such as wastewater treatment, water–salt separation and sea resource production.

## Teaser

A 3D interconnected periodic carbon-tube membrane with an interfacial water layer on top enables self-cleaning solar brine treatment and autonomous salt production by balancing gravity, buoyancy and surface tension.

## INTRODUCTION

Given the abundance of valuable resources in water sources such as the sea, lakes and even wastewater, persistent efforts have been made to separate solutes from water, enabling the recovery of both resources and water [1–3]. As an example, water and salt, which play the most vital roles in the daily life of plants, animals and human beings, can both be harvested from the sea. However, the natural solar evaporation process remains highly inefficient, requiring large areas with stringent irradiation and temperature conditions.

**Figure 1.**
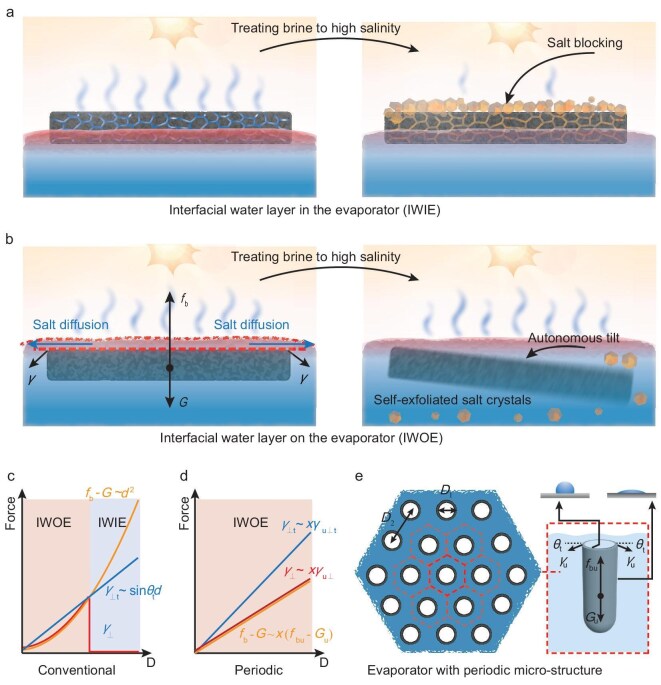
Schematics of solar water treatment by interfacial evaporation. (a) Conventional evaporator with interfacial water layer existing within it (IWIE). It will suffer from a salt-clogging problem during the treatment of high-salinity brine. (b) Unique evaporator with interfacial water layer existing on top of it (IWOE). When high-salinity brine is treated, the membrane can spontaneously tilt and dump the precipitated salt crystals, realizing self-cleaning and autonomous salt production. (c, d) Variation trend of the force difference (${f_{\mathrm{b}}} - G$), the threshold of the vertical component of the surface tension (${\gamma _ \bot }$_t_) and the actual vertical component of the surface tension (${\gamma _ \bot }$) with the size increment for (c) a conventional evaporator and (d) the evaporator with a periodic microstructure. When the force difference is smaller than the threshold of the vertical component of the surface tension, the evaporator remains balanced in the water, maintaining the IWOE state. Once ${f_{\mathrm{b}}} - G$ exceeds ${\gamma _ \bot {\rm t}}$, the evaporator will float above the water interface, entering the IWIE state. (e) Scalable evaporator consisting of periodic tube arrays. Each tube unit reaches the equilibrium of gravity (${G_{\mathrm{u}}}$), buoyancy (${f_{{\mathrm{bu}}}}$) and surface tension (${\gamma _{\mathrm{u}}}$) under the water.

Recently, interfacial solar vapor generation has provided a promising alternative for water treatment, with green energy and minimized carbon footprint [4–21]. However, in most of the previously reported interfacial solar desalination processes, the interfacial water layer typically exists within the solar evaporator (namely an ‘interfacial water layer in the evaporator’, denoted as IWIE) [22–25]. As shown in Fig. 1a (left), when brine with high salinity is treated, salt crystals precipitate and adhere to the surface or channels of the evaporator after continuous evaporation (Fig. 1a, right). The accumulated salt precipitates shade incident light or destroy the internal structures of the evaporator, significantly degrading the performance and limiting the

working life span [26]. Most, if not all, of these antifouling designs can be divided into two strategies: salting-out and salting-free [3,10,27–31]. While salting-free strategies are completely unaffected by salt precipitation, they are mostly applicable to treating brine with moderate concentrations and will degrade when treating saturated brine. Salting-out strategies can treat brine with various concentrations, but these designs (without a filmwise water layer on the evaporators) enable salt to precipitate easily and salt tends to stick on the evaporator surfaces. It is still challenging to postpone the salt precipitation and realize self-cleaning simultaneously with one device during the whole water-treatment process.

In this work, we propose the concept of an interfacial water layer on top of a solar evaporator (namely an ‘interfacial water layer on the evaporator’, denoted as IWOE). This design takes advantage of the strengths of both the salting-free and salting-out strategies. The interfacial water layer is heated locally under solar illumination for fast evaporation, while also serving as salt-diffusion layer for postponing the salt-precipitation process (Fig. 1b, left). When the brine reaches saturated salinity, the salt nucleates and grows within the interfacial water layer rather than adhering to the evaporator. As the growing crystals disturb the mechanical balance, the evaporator tilts and the non-adherent salt crystals glide easily, realizing the self-cleaning process autonomously (Fig. 1b, right).

This unique working state of IWOE takes advantage of the surface tension $( \gamma )\,\,$at the air/water/evaporator interface. When the evaporator is initially immersed in water, it floats up and touches the air/water interface. This two-phase interface will be slightly distorted and form an air/water/evaporator three-phase interface, loading surface tension on the underwater evaporator. The vertical component of the surface tension (${\gamma _ \bot }$) tends to overcome the force difference between buoyancy and gravity (${f_{\mathrm{b}}} - G$) and causes the evaporator to reach mechanical equilibrium below the air/water interface.

However, as $({f_{\mathrm{b}}} - G)$ and ${\gamma _ \bot }$ often increase with different trends along with the increment of the evaporator size, it is difficult to maintain equilibrium between gravity, buoyancy and surface tension as the evaporator scales up. For a conventional evaporator with fixed thickness, buoyancy and gravity are proportional to the area (Equations (1) and (2)). Differently from that, the vertical component of the surface tension is only proportional to the perimeter and has a threshold related to the wetting property of the edge (Equation (3)) [32–34]:


(1) \begin{eqnarray*}
{f_{\mathrm{b}}} = \frac{\pi }{4}{\rho _{\mathrm{w}}}{d^2}h {\mathrm{\,\,}} \sim {\mathrm{\,\,}} {d^2},
\end{eqnarray*}

(2) \begin{eqnarray*}
G = \frac{\pi }{4}{\rho _{\mathrm{e}}}{d^2}h{\mathrm{\,\,}}\sim {\mathrm{\,\,}}{d^2},
\end{eqnarray*}

(3) \begin{eqnarray*}
{\gamma _{ \bot {\mathrm{t}}}} = sin{\theta _{\mathrm{t}}}\gamma = sin{\theta _{\mathrm{t}}}\sigma \pi d{\mathrm{\,\,}}\sim {\mathrm{\,\,}} sin{\theta _{\mathrm{t}}}d,
\end{eqnarray*}

where ${\rho _{\mathrm{w}}}$ and ${\rho _{\mathrm{e}}}$ denote the density of the water and the evaporator, respectively; *d* and *h* represent the diameter and thickness of the evaporator; and ${\theta _{\mathrm{t}}}$ represents a threshold angle between the direction of surface tension and the horizontal line, which is positively related to the hydrophilicity of the edge of the evaporator. As shown in Fig. 1c, the surface tension can balance the force difference for the evaporator with a small size, achieving the state of IWOE. However, as the evaporator scales up, the force difference exceeds the threshold of the surface tension. In this case, the evaporator will float upon the water interface, changing the state to IWIE, and will no longer be affected by the surface tension.

Thus, to keep the IWOE state regardless of the evaporator scale, the evaporator needs to be elaborately designed with scalable periodic building blocks (Fig. 1d) and each block should have the correct density and hydrophilic periphery, meeting the requirement of Equation (4):


(4) \begin{eqnarray*}
{f_{{\mathrm{bu}}}} - {G_{\mathrm{u}}} = {\gamma _{{\mathrm{u}} \bot }} \le {\gamma _{{\mathrm{u}} \bot {\mathrm{t}}}},
\end{eqnarray*}

where ${f_{{\mathrm{bu}}}}$ and ${G_{\mathrm{u}}}$ represent the buoyancy and gravity of each block; and ${\gamma _{{\mathrm{u}} \bot {\mathrm{t}}}}$ and ${\gamma _{{\mathrm{u}} \bot }}$ represent the threshold and the actual value of the surface tension loaded on each block, namely the vertical component of the surface tension. Due to the periodicity of the evaporator, the total force difference (${f_{\mathrm{b}}} - G$) and vertical component of the surface tension (${\gamma _{ \bot {\mathrm{t}}}}$ and ${\gamma _ \bot }$) are increased with the same trend as the scaling-up (Equations (5) and (6)). Therefore, the evaporator can perfectly maintain the IWOE working state (Fig. 1d):


(5) \begin{eqnarray*}
{f_{\mathrm{b}}} - G{\mathrm{\,\,}} \sim {\mathrm{\,\,}}x\left( {{f_{{\mathrm{bu}}}} - {G_{\mathrm{u}}}} \right) = x{\gamma _{{\mathrm{u}} \bot }},
\end{eqnarray*}

(6) \begin{eqnarray*}
{\gamma _{ \bot {\mathrm{t}}}}{\mathrm{\,\,}}\sim {\mathrm{\,\,}}x{\gamma _{{\mathrm{u}} \bot {\mathrm{t}}}},
\end{eqnarray*}

where *x* represents the assumed number of the repeating building blocks.

In this work, we experimentally demonstrate the concept of IWOE by using an elaborately designed 3D integrated periodic carbon-tube (CT) membrane (denoted as 3D-CT). The 3D-CT membrane consists of hexagonally arranged periodic vertical CTs with a tailored CT-diameter (*D*_1_) and inter-CT distance (*D*_2_) between the neighboring vertical CTs, as well as hydrophilic external and hydrophobic internal tube surfaces (Fig. 1e). The hydrophobic internal surfaces cause each tube to be filled with air to obtain the correct overall gravity and buoyancy, and the hydrophilic external surfaces generate downward surface tension on each tube. As each tube unit reaches equilibrium between gravity, buoyancy and surface tension under the water, the total evaporator can maintain the IWOE state with a scalable size (Fig. 1e). The naturally existing interfacial water layer is locally heated under solar illumination, featuring an enhanced solar evaporation rate. When high-salinity brine is treated, the membrane spontaneously tilts and dumps the precipitated salt crystals, realizing the self-cleaning property. Meanwhile, the salt crystals sink to the bottom and can be easily collected and recovered at any time.

## RESULTS AND DISCUSSION

### Fabrication and characterization of the periodic 3D-CT membrane

**Figure 2.**
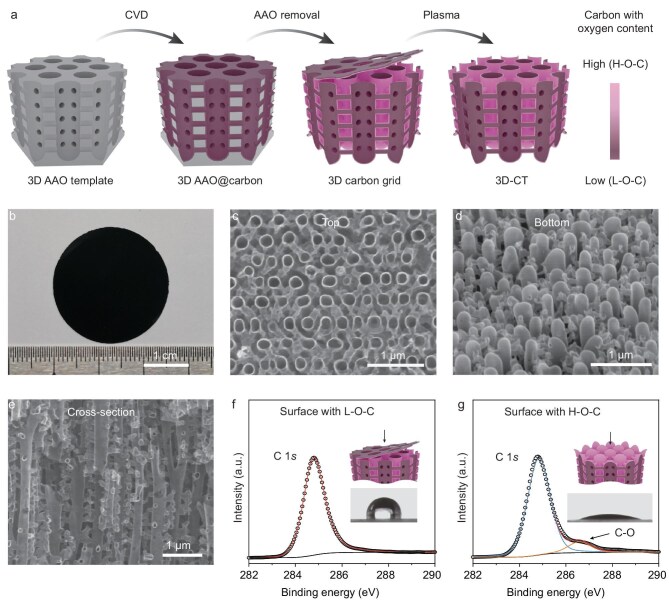
Fabrication and characterization of the 3D-CT membrane with each vertical tube’s top end open and bottom end closed. (a) Schematic fabrication process for the 3D-CT membrane. (b) Optical photograph of the 3D-CT membrane. (c–e) Highly magnified SEM images of the (c) top, (d) bottom and (e) cross-sectional views of the 3D-CT membrane, respectively. (f, g) X-ray photoelectron spectroscopy (XPS) spectra and contact angles of surfaces consisting of carbon with (f) low oxygen content (L-O-C) and (g) high oxygen content (H-O-C), respectively.

The periodic microstructure, surface chemistry and underwater density are all critical features of the evaporator and should be carefully designed. To obtain an evaporator with highly periodic and robust microstructures without scattering or clustering when immersed in water, a 3D interconnected nanoporous template with hexagonally arranged vertical nanochannels interconnected by lateral narrow nanochannels is adopted [35]. The fabrication process mainly comprises steps of the chemical vapor deposition (CVD) of carbon on the inner pore walls of the 3D interconnected nanoporous template, subsequent selective template removal and Ar plasma treatment (Fig. 2a). The 3D interconnected nanoporous anodic aluminum oxide (AAO, 3D-AAO) template used here helps in the formation of the 3D interconnected periodic CT-based structure. In addition, the AAO template also brings about discrepancies in the oxygen content (as well as the wetting properties) of the surfaces of each CT, causing them to have an internal-air/external-water filling state under the water to achieve enough buoyancy. The oxygen in the AAO template can diffuse to the periphery of the vertical CTs during the high-temperature carbon condensation, providing a convenient method for hydrophilizing the periphery (outside surface of each CT unit) while keeping its inner surface hydrophobic. Through selective removal of the AAO via alkaline etching, a 3D carbon grid with inhomogeneous wetting properties is obtained. After Ar plasma is used to remove the top carbon layer, the specially designed highly stable periodic 3D-CT membrane with each vertical tube’s top end open and bottom end closed is finally obtained. When an AAO template with a larger area is utilized, the 3D-CT membranes can be scaled up to a larger size (Fig. S1). As the 3D-CT is tailored to have the proper size and distributions and the inhomogeneous wetting properties of carbon walls, each CT unit can achieve equilibrium between gravity, buoyancy, and surface tension realizing the desired IWOE state.

The 3D-AAO template (Fig. S2) is prepared by anodizing specific aluminum (Al) foil with Cu impurities in phosphoric acid solutions [36]. Figure S3 shows the scanning electron microscopy (SEM) image of the 3D carbon grid after removal of the 3D-AAO template. After subsequent removal of the top carbon layer of the CTs, the 3D-CT membrane with each vertical tube’s top end open and bottom end closed is obtained and its optical image is shown in Fig. 2b. SEM images of the top, bottom and cross-sectional views of the 3D-CT membrane are demonstrated in Fig. 2c–e, respectively. It can be seen that the thicker CTs with diameters of 200–300 nm are vertically aligned and uniformly distributed hexagonally, with central distances of 400–500 nm (Fig. 2c and d), while the lateral thinner CTs with diameters of 50–80 nm interconnect the neighboring vertical thicker CTs together into an integrated and robust 3D-CT membrane (Fig. 2e). Optical measurement shows that the 3D-CT membrane exhibits an ultrahigh broadband absorption of ∼99% weighted by the standard solar spectrum (AM 1.5 G) (Fig. S4), showing great potential for solar thermal conversion.

The carbon surfaces exhibit inhomogeneous wetting properties due to the inhomogeneous oxygen content (Fig. 2f and g). For the carbon surface touching the AAO template (such as the bottom side of the 3D carbon grid and periphery of each CT), the oxygen content is significantly increased due to oxygen diffusion at a temperature of 650°C during the CVD process (Fig. 2g). This area of carbon has high oxygen content, denoted as H-O-C, whereas the top side of the 3D carbon grid and the internal surface of each CT are away from the AAO template and have low oxygen content (Fig. 2f and Fig. S3), denoted as L-O-C. The H-O-C and L-O-C surfaces have very different wetting properties. As a result, the bottom surface of the 3D carbon grid (with H-O-C) has a small water-contacting angle of 21°, while the top surface (with L-O-C) has a large water-contacting angle of 102°. These inhomogeneous wetting properties together with the top-end-open and bottom-end-closed configurations are fairly significant for achieving the correct gravity and buoyancy underwater, which will be discussed later.

### IWOE working state for the 3D-CT membrane

**Figure 3.**
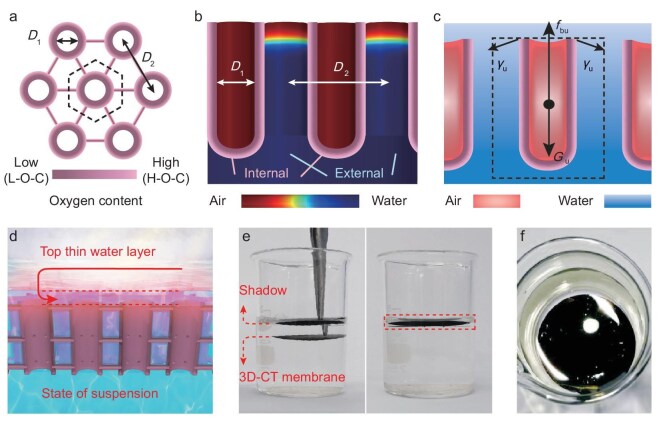
Demonstration and mechanical analysis of the IWOE working state of the 3D-CT membrane. (a) Schematic top view of the 3D-CT membrane, in which the external and internal tube surfaces possess different oxygen contents and thus different wetting properties. *D*_1_ denotes the diameter of the vertical CT, which is 200–300 nm; *D*_2_ denotes the central distance of adjacent vertical CTs, which is 400–500 nm. (b) COMSOL-simulated internal-air/external-water filling states for CTs. (c) Mechanical analysis of the 3D-CT membrane under water. ${G_{\mathrm{u}}}$, ${f_{{\mathrm{Bu}}}}$ and ${\gamma _{\mathrm{u}}}$ denote the gravity, buoyancy and surface tension imposed on a vertical CT, respectively. It reaches the equilibrium of buoyancy, gravity and surface tension. (d) Schematic of the IWOE state of the 3D-CT membrane. (e) Optical photographs showing that the 3D-CT membrane exhibits the IWOE state underwater. (f) Shining surface of the 3D-CT membrane in the IWOE state.

The different oxygen contents (H-O-C and L-O-C for external and internal CT surfaces) and resulting different wetting properties (contacting angles of 21° and 102° for H-O-C and L-O-C surfaces) are the key points for the internal-air/external-water filling state of the 3D-CT membrane (Fig. 3a). This can be verified by the results of COMSOL Multiphysics simulations (Fig. 3b). The lateral thinner CTs, used for integrating the thicker vertical CTs together, are neglected in COMSOL simulation and mechanical analysis. This is because lateral CTs with diameters that are less than one order of magnitude of the vertical CTs are considered not to change the mechanical properties of the 3D-CT membrane. The COMSOL simulation results show that water cannot enter the inner cavity of CTs with diameters of 200–300 nm and hydrophobic internal surfaces with L-O-C (contacting angle of ∼102°) [34,37,38], whereas, for spaces outside the CTs with hydrophilic surfaces (with H-O-C, contacting angle of ∼21°), water can easily enter and fill the space through capillary force [34,37,38]. The simulated water filling condition is shown in Fig. 3b and the simulation details can be found in the ‘Materials and methods’ section in Supplementary Information.

To further understand the IWOE working state of the 3D-CT membrane with a periodic structure, a vertical CT as a unit is used for the subsequent mechanical analysis. As shown in Fig. 3c, the gravity (${G_{\mathrm{u}}}$) and buoyancy (${f_{{\mathrm{bu}}}}$) applied to each CT are calculated as follows:


(7) \begin{eqnarray*}
{G_{\mathrm{u}}} = {m_0}hg,
\end{eqnarray*}

(8) \begin{eqnarray*}
{m_0} = {\rho _{\mathrm{c}}}\pi {D_1}\Delta D,
\end{eqnarray*}

(9) \begin{eqnarray*}
{f_{{\mathrm{bu}}}} = \frac{\pi }{4}D_1^2h{\rho _w}g,
\end{eqnarray*}

(10) \begin{eqnarray*}
{\gamma _{{\mathrm{u}} \bot {\mathrm{t}}}} = \pi {D_1}\gamma \sin {\theta _{\mathrm{t}}},
\end{eqnarray*}

where *h* denotes the thickness of the membrane, namely the length of each CT, which is 30 μm;$\,\,{\rho _{\mathrm{c}}}\,\,$represents the density of carbon, which typically ranges from 1.8 to 2.3 g cm^−3^; *D*_1_ denotes the diameter of the CT, which is 200–300 nm; $\Delta D$ represents the thickness of the wall of CTs, which ranges from 5 to 10 nm (Fig. S5);$\,\,g$ represents the acceleration of gravity (9.8 m s^−2^); $\gamma $ represents the surface tension of the solution, which is 0.073 N m^−1^; and ${\theta _{\mathrm{t}}}$ represents the contact angle of the membrane surface, which is 21° for the CT.

The membrane that is totally underwater has ${f_{{\mathrm{bu}}}} > {G_{\mathrm{u}}}$ for each CT. The upward net force $( {{f_{{\mathrm{bu}}}} - {G_{\mathrm{u}}}} )\,\,$will drive the membrane up to the water surface and then surface tension (${\gamma _{\mathrm{u}}}$) is exerted on the hydrophilic external surface of each CT. Hence, each CT reaches mechanical equilibrium just below the water surface, suspending the 3D-CT membrane in a state of IWOE (Fig. 3d). As each unit achieves mechanical equilibrium, it is expected that the IWOE state can be maintained with a scaling-up of the membrane. Furthermore, the lateral CTs, which serve as interconnecting linkages, enhance the robustness of the 3D‑CT membrane by enabling it to behave as a collective system. Even if a small number of the tubes lose air entrapment (due to wall defects or partial wetting of internal tube walls), the resultant loss in local buoyancy can be compensated for by the upward force provided by surrounding intact tubes (Note S1).

Figure 3e shows an optical photograph of the 3D-CT membrane in an IWOE state. It will not sink even after being pressed into the deep water. The shining surface shown in Fig. 3f also indicates the existence of the interfacial water layer above the membrane that is totally different from that of the 3D carbon grid (before removal of the top carbon layer), which floats on the water surface, as it fails to form a periodic structure in water (Fig. S6). The IWOE working state of the membrane is governed by the gravity, buoyancy and surface tension of the membrane underwater, which are intrinsic to the structure and wetting properties of the membrane. These characteristics are inherent and thus remain stable regardless of the external environmental conditions. As a result, the 3D-CT membrane can reliably maintain its IWOE state in diverse environments, including wind, waves and thermal fluctuations (Note S2).

To further prove the vital role of the adverse wetting properties of the CT walls (hydrophilic for the external surface with H-O-C, hydrophobic for the internal surface with L-O-C) for the IWOE state, ethanol is added into the water to reduce the surface tension and enhance the wetting property. The contact angle of the L-O-C surface reduces to ∼66° when using 20 wt% ethanol solution, suggesting that both the external and internal CT walls become hydrophilic in 20 wt% ethanol solution. As expected, the 3D-CT membrane immediately sinks to the bottom when ethanol is added to the water (Fig. S7). Thus, it is believed that the adverse wetting property is critical for realizing the IWOE state.

These results also indicate the need to consider water composition in practical applications. The IWOE-based membrane can perform reliably in saline wastewater and seawater, in which the wetting properties remain largely unaffected. For wastewater containing surfactants or organic contaminants, which can lower the surface tension and diminish the wettability contrast, further adaptations would be required. These could include pretreatment to remove surfactants or redesigning the density and surface wettability of the membrane to maintain mechanical equilibrium under the reduced surface tension.

### Performance of solar evaporation by the 3D-CT membrane

**Figure 4.**
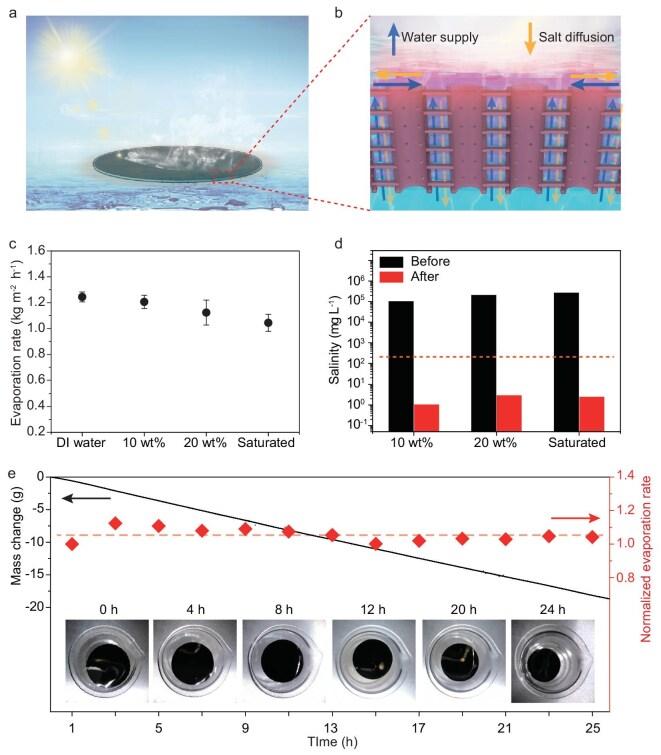
Performance of solar vapor generation. (a, b) Overall and detailed schematic of the interfacial water layer that induced solar evaporation based on a 3D-CT membrane. (c) Evaporation rates of 3D-CT membranes for different water resources. (d) Salinities of different brine samples (10 wt%, 20 wt% and saturated) before and after water purification. The dashed line refers to the World Health Organization (WHO) standards for drinkable water. (e) Stable solar water treatment of brine with 10 wt% as the initial salinity.

The 3D-CT membrane in an IWOE working state offers several unique advantages to enable the efficient solar water treatment of high-salinity brine (Fig. 4a). Besides ultrahigh (∼99%) solar absorption for effective energy input, the naturally existing interfacial water layer above the 3D-CT membrane is locally heated for effective evaporation (Fig. 4b). In addition, owing to the good hydrophilicity of the internal surface of each CT, it can be calculated that the water is supplied very quickly at an average velocity of 20 m s^−1^ in such nanochannels with diameters of 400–500 nm (Fig. S8), which is sufficiently high for fast evaporation. As the salt concentration of the thin water film increases (but does not reach the saturated state) with water evaporation, the salt diffuses downwards, away from the 3D-CT membrane, postponing the salt-precipitation process.

The 3D-CT membrane demonstrates good solar desalination performance for treating various water resources (deionized (DI) water, brine water with 10 and 20 wt% salinity, and saturated brine water with ∼26.5 wt% salinity). The mass changes of all water resources under 1-sun illumination are demonstrated in Fig. S9. The evaporation rates of DI water, brine water with 10 and 20 wt% salinity, and saturated brine water were measured to be 1.24, 1.21, 1.12 and 1.04 kg m^−2^ h^−1^, respectively, as summarized in Fig. 4c. The slower evaporation for brine with higher salinity may be ascribed to the enhanced enthalpy of the phase change. The heat-loss analysis of solar evaporation based on the 3D-CT membrane is also demonstrated in Note S3. The effect of solar desalination for the 3D-CT membrane is also systematically evaluated by carefully tracking the ion concentrations in purified water. A transparent condensation device is used to collect the purified water (Fig. S10). It is found that the salinities are significantly reduced (by five orders of magnitude) after solar water treatment (Fig. 4d), which can meet the World Health Organization standard for drinking water [39]. Similarly, the concentrations of all the primary ions (Na^+^, Mg^2+^, Ca^2+^ and B^3+^) in seawater collected from the Bohai Sea also dramatically decreased after desalination (Fig. S11).

As expected, the 3D-CT in an IWOE working state can avoid performance degradation and fouling issue when treating unsaturated brine—a behavior consistently supported by COMSOL simulations of evaporation‑driven and diffusion-driven salt fluxes (Note S4). Here, we performed a continuous 25-hour experiment of solar desalination under a solar simulator. As shown in Fig. 4e, during the continuous solar desalination under 1-sun illumination for 25 hours, the mass change in the brine water (10 wt% as the initial salinity) with the 3D-CT membrane evolved almost linearly. The normalized evaporation rates during the desalination process were calculated, as demonstrated in Fig. 4e, indicating that the performance of the 3D-CT membrane remains stable when treating high-salinity brine. It is also observed that the 3D-CT membrane remains clean, without salt precipitation during this process, exhibiting good anti-salt properties. Moreover, the 3D-CT membrane can also realize salt-resistant solar desalination when treated with real seawater from the South China Sea, as shown in Fig. S12. The membrane maintains its original structure and periodicity without structural creep after the continuous solar treatment of seawater (Fig. S13).

In addition to the stability of anti-salt solar evaporation, the 3D-CT membrane also demonstrates material and structural stability, as it is composed of carbon materials with strong chemical bonds. To assess the material and structural stability of the 3D-CT membranes, they were immersed in 25 wt% NaCl solutions and 3 M H_2_SO_4_ at 90°C for 7 days. These conditions represent extreme scenarios encountered in brine treatment or wastewater treatment. The SEM images show that the membrane maintains the same nanostructure without any aging or corrosion after the treatment, as shown in Fig. S14. Transmission electron microscopy (TEM) images also demonstrate good stability of the 3D-CT membranes, as it can be seen that the carbon walls of the 3D-CT remain smooth after the treatment of NaCl solutions and 3 M H_2_SO_4_ at 90°C (Fig. S15).

### Self-cleaning and salt-producing processes of the 3D-CT membrane

**Figure 5.**
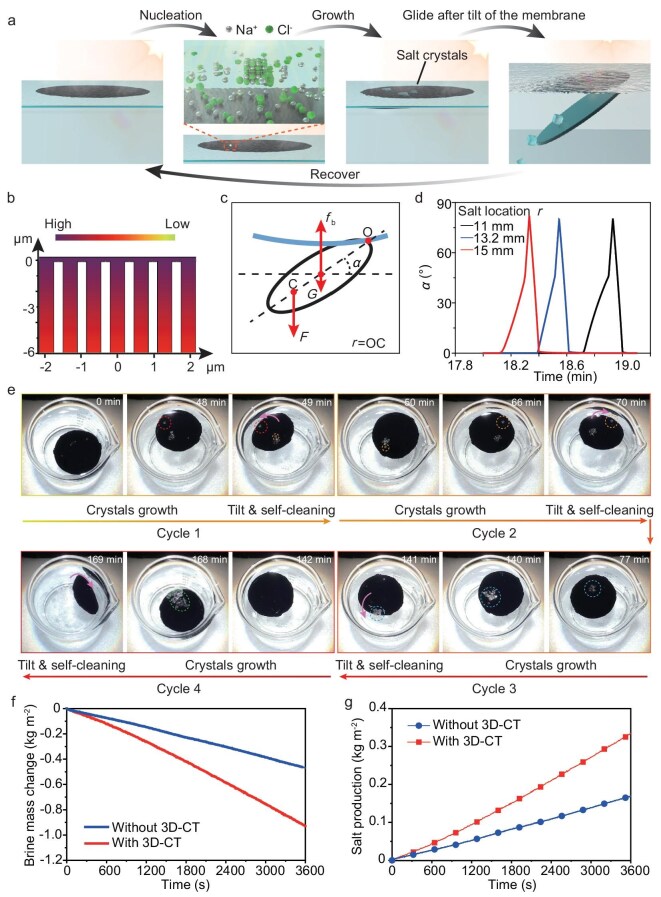
Self-cleaning and salt-producing process of the 3D-CT when treating saturated brine. (a) Schematic of the self-cleaning and salt-producing process. (b) Simulated salt distribution of the 3D-CT membrane. The interfacial water layer makes it easier to reach the crystallization concentration. (c) Mechanical analysis of the process. (d) Simulated movement of the 3D-CT. (e) Repeated self-cleaning and salt-producing process of the 3D-CT when treating saturated brine. (f) Mass changes and (g) salt-producing performances of the saturated brine with and without the 3D-CT membrane over time under 1-sun illumination.

The interfacial water layer on the 3D-CT membrane endows it with abilities of self-cleaning and autonomous salt production when treating highly concentrated or even saturated brine (Fig. 5a). This is because the salt nucleates and grows within the interfacial water film on top of the solar evaporator rather than adhering to the evaporator, and can glide easily after the evaporator is tilted, realizing self-cleaning and autonomous salt-production processes.

The salt tends to nucleate within the interfacial layer due to the increasing salt concentration in the system. As simulated by using COMSOL (Fig. 5b), the top water layer exhibits a higher salt concentration compared with that of the water in the interspaces of the CTs. Thus, it is expected that the interfacial water layer makes it easier to reach the critical crystallization concentration after continuous solar evaporation.

Here, we perform a mechanical analysis of the 3D-CT membrane in the IWOE state, as salt nucleates and grows during the solar water treatment of saturated brine (Fig. 5c and d). While each vertical CT maintains a local balance between gravity, buoyancy and surface tension, the lateral thinner CTs integrate them into a monolithic, rigid 3D-CT membrane for mechanical analysis. The salt crystals grow randomly, loading a resultant force *F* at the C point on the 3D-CT membrane. The C point denotes the mass center of the salt crystals and *F* is determined by the gravity and buoyancy of the salt crystals. *F* increases with the growth of the salt crystals, loading an increased torque on the membrane, which eventually results in tilting of the membrane and sliding of the salt crystals. The pivot point O is located at the edge of the membrane, which provides the biggest torque $( {F \times r} )$ for the tilt (*r* is the distance between O and C).

For simulating the tilt process, we calculate the curves of the inclination angle *α* over time for the situations with salt crystals precipitating at different locations (with different *r*) on a 3D-CT membrane with a diameter of 2 cm (Fig. 5d). It shows that the suspended 3D-CT membrane does not exhibit obvious tilt within the first ∼20 min, during the growth of salt crystals. Once the increasing *F* overcomes the force difference between buoyancy and gravity, the membrane detaches from the water film and the tilt of the membrane is initiated at an appreciable rate. The membrane tilts at a faster rate to return to the original IWOE state after the gliding of the salt crystals. This self-cleaning process (the tilting of the 3D-CT membrane and the gliding of the salt crystals) is completed within only ∼0.5 min and the precipitated salt crystals are produced and can be collected at the bottom. Notably, although the nucleation of salt is a stochastic process, leading to variability in the precipitation locations across the cycles, the subsequent self-cleaning mechanism remains highly reproducible and mechanically deterministic (Supplementary Note S5). Besides, the open tops of the carbon tubes do not affect the buoyancy or IWOE recovery during the self‑cleaning tilt. This is because the top‑open and bottom‑closed geometry and hydrophobic internal surface together preserve entrapped air within the tubes, even when the tube openings briefly approach the air–water interface, thus ensuring stable buoyancy and reversible IWOE recovery.

The repeated self-cleaning and salt-producing process of the 3D-CT membrane when treating saturated brine under 1-sun illumination is recorded in Video S1. Typical optical images are demonstrated in Fig. 5e, revealing the process of crystal growth, tilt, self-cleaning and salt production of the membrane. Taken together, the intervals of the adjacent self-cleaning process ranged from ∼70 to 120 min. This is longer than that in the estimation, as the *F* value is smaller in the real situation due to the adhesion between the salt crystals and the water surface. It can be observed that the self-cleaning process is fast, within 1 min, which is consistent with the analysis and prediction presented in Fig. 5d. The repeated observation that salt crystals grow and then glide off the membrane surface during the self-cleaning cycle also suggests that the crystals are not anchored to solid surface features. As the top-suspending 3D-CT membrane enables an evaporation rate that is approximately twice that for saturated brine without the 3D-CT (0.505 kg m^−2^ h^−1^) (Fig. 5f), the calculated salt-production rate can also be doubled by utilizing the 3D-CT membrane in the IWOE working state (Fig. 5g).

## CONCLUSION

In summary, we propose the concept of an interfacial water layer on top of the evaporator (IWOE) for design principles, as it enables self-cleaning solar brine treatment and autonomous salt production. Further, we experimentally demonstrate that a periodic 3D-CT membrane with well-tailored nanostructures and inhomogeneous wetting properties is capable of suspending on the water surface with the natural existence of the interfacial water layer above, taking advantage of the elegant equilibrium between gravity, buoyancy and surface tension. This membrane not only realizes efficient solar water treatment, but also autonomously self-cleans and produces salt at the bottom by adjustment of the mechanical balance. Nevertheless, it is important to recognize that the 3D-CT membrane is primarily a proof of concept for IWOE. In consideration of scalability, future work will focus on developing low-cost materials and commercially viable configurations based on the IWOE concept. It calls for alternative material systems that realize the essential mechanical balance between gravity, buoyancy and surface tension, yet are simpler and more economical to produce. Examples include porous polymers or carbon‑based substrates with tailored surface chemistry and broadband optical coatings, which will improve manufacturability and cost‑effectiveness (Note S6). We anticipate that such advances will enable the practical deployment of IWOE‑based systems in scalable wastewater treatment, water–salt separation and sea resource production, thereby extending the impact of this interfacial evaporation strategy beyond the laboratory.

## Supplementary Material

nwag188_Supplemental_Files
