# Supplementary material for: 3D interconnected periodic carbon-tube membrane enabled self-cleaning solar brine treatment and autonomous salt production
Source: Natl Sci Rev. 2026 Apr 6;13(10):nwag188. doi: 10.1093/nsr/nwag188 (PMC13192534; doi:10.1093/nsr/nwag188)
Supplement: nwag188_Supplemental_Files [file nwag188_supplemental_files.zip › Supplementary Information.pdf]

Supplementary Information for

3D interconnected periodic carbon-tube membrane enabled self-cleaning solar brine treatment and autonomous salt production

Ning Xu, Qijun Pan, Chun Shen, Ou Qian, Shiqi Fang, Fangming Han, Mengyue Zeng, Songguo Li, Jia Zhu, Wanlin Guo, Guowen Meng

Corresponding author: gwmeng@issp.ac.cn; wlguo@nuaa.edu.cn; jiazhu@nju.edu.cn;

Methods

Fabrication of 3D-AAO template

The 3D-AAO templates used were prepared via Al foil anodization and subsequent selective etching process. First, in a mixture solution (0.3 M H_3PO_4, H_2O: C_2H_6O=9:1 in volume) at 0ºC, Al foil with Cu impurities (99% purity, 100 μm thick) was anodized at a constant voltage of 185 V for 15 h. After removing the remaining bottom Al foil in a saturated $SnCl_4$ solution, the sample was immersed in 5 wt% H_3PO_4 at 40ºC for 25 min to selectively etch the Cu impurity nanoparticles embedded in the vertical nanopore walls and widen the pores to obtain the nanoporous 3D-AAO template.

Fabrication of the periodic 3D-CT membrane

The periodic 3D-CT membrane was fabricated by the 3D-AAO template confined growth in a CVD process at ambient pressure and 650ºC for 2 h with a flow of the gas mixture of 80 standard cubic centimeters per minute (sccm) of Ar and 6 sccm of C_2H_2. The sample (3D-AAO template embedded with 3D carbon grid) was then cleaned by Ar plasma with a Plasma Cleaner (Harrick Plasma, PDC-32G) for 10 min to remove the surface amorphous carbon of barrier layer of AAO, and then immersed in a 3 M NaOH solution at 60ºC for 5 h to selectively remove the AAO template to achieve the 3D carbon grid. Finally, the top surface of 3D carbon grid was cleaned by Ar plasma with the same Plasma Cleaner as above for 30 min to remove the top carbon layer to obtain top-end-open and bottom-end-closed 3D-CT membrane. In the fabrication process, uniformity and controllability are achieved through a combination of real-time monitoring and meticulous process design. Firstly, to ensure uniform reaction temperature, the 3D-AAO template is placed in the middle of the quartz tube. Meanwhile, each layer of the sample is separated by the porous foamy copper, which serves to prevent the blockage of nanopores and to minimize positional movement during batch processing. Secondly, the gas flow rate is precisely monitored and controlled through the mass flow meter in real-time. After the CVD reaction, the unreacted acetylene can be promptly evacuated by mechanical pump. Consequently, the carbon wall thickness can be effectively controlled by adjusting the CVD reaction time.

Experimental setup for solar desalination

A solar simulator (94043A, Newport), equipped with an optical filter for the standard AM 1.5 G spectrum, was used as the illuminant for all indoor experiments of solar desalination and wastewater treatment during the experiments, the indoor temperature and humidity were carefully controlled to ~28°C and ~40%, respectively.

Evaluation of the evaporation performance

The mass change of water, used for determining the evaporation rate, was real-time measured by a high accuracy balance (FA 2004, 0.1 mg in accuracy). The evaporation rate was obtained by calculating the slope of the curve of mass change at a steady-state (3000 s-3600 s).

Characterizations

The microscopic structures of the 3D-CT membrane were characterized by SEM (FEI Helios Nanolab 600i) and TEM (Tecnai G2 F20). The oxygen contents in carbon surfaces were measured by XPS (ESCALAB 220i-XL). The absorption spectra of the evaporators were measured by UV/vis spectroscopy (UV-3600, Shimadzu) attached to an integrating sphere (ISR-3100). A surface tension-contact angle meter (GBX Digidrop) was used to measure the water contact angle of the sample. The concentrations of cations were monitored by ICP-OES (PerkinElmer Instruments, PTIMA 5300 DV). The infrared images of the evaporator surface were captured by an infrared camera (FLUKE, TiX 580).

COMSOL simulations

Water filling state inside the 3D-CT membrane is simulated using the COMSOL package. The two-phase flow phase filed and laminar flow interfaces are selected to describe the flow of water and air. Hollow cylinders with diameters of 250 nm and 400 nm are constructed to mimic the internal and external space of the CTs in the 3D-CT, respectively. The length of the cylinders is 800 nm. Besides, a disk with diameter of 800 nm and thickness of 200 nm is constructed to mimic the bulk water, which connects with the CTs. The model uses the predefined wetted wall boundary condition at the solid walls, with contact angles of hydrophilic 20° for internal CT walls and hydrophobic 102° for external CT walls. The surface tension of water is 0.079 N m^{-1}.

Supplementary Figures

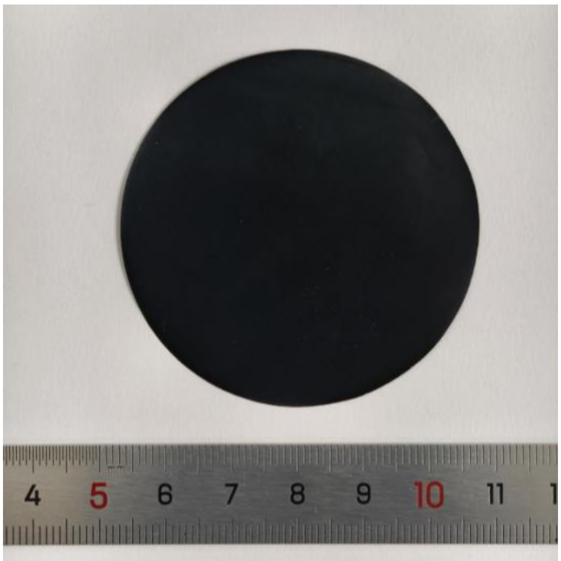

Figure S1. The photograph of the 3D-CT membrane on a large scale.

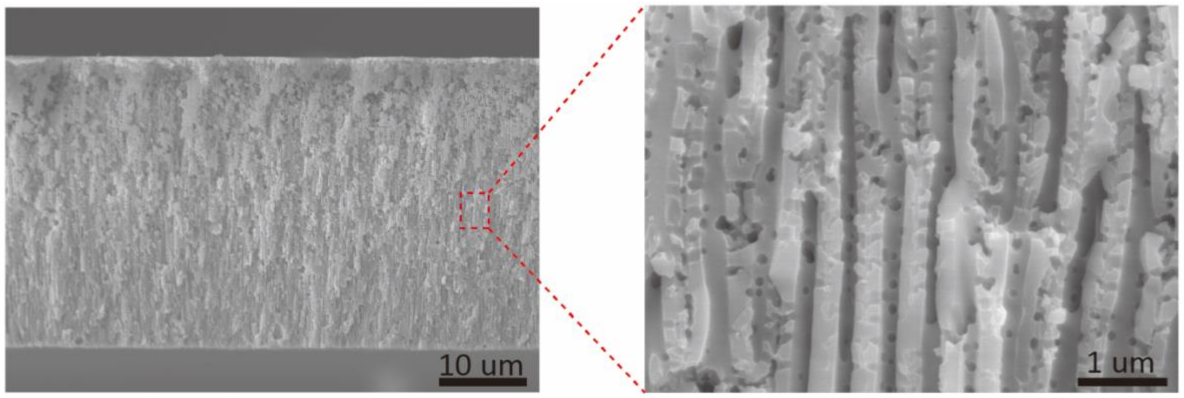

Figure S2. SEM images of the 3D interconnected nanoporous anodic aluminum oxide (AAO). Vertical nanochannels and lateral thinner nanochannels can be observed in the AAO membrane.

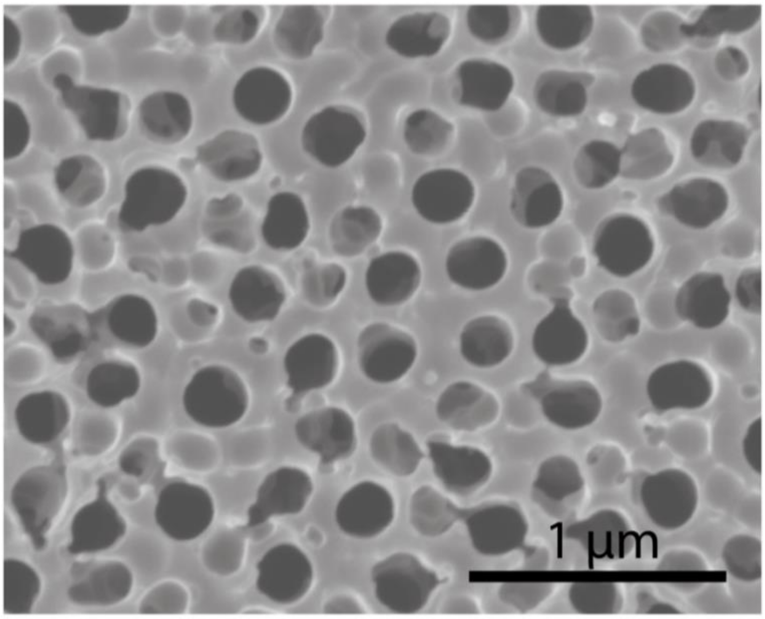

Figure S3. SEM images of the top views for the 3D carbon grid. The top surface of the 3D carbon grid consists of carbon with low oxygen content (L-O-C), and exhibits a water contact angle of ~102 º (fig. 2f). Differently, the bottom surface of the 3D carbon grid consists of carbon with high oxygen content (H-O-C), and exhibits a water contact angle of 21 º (fig. 2g).

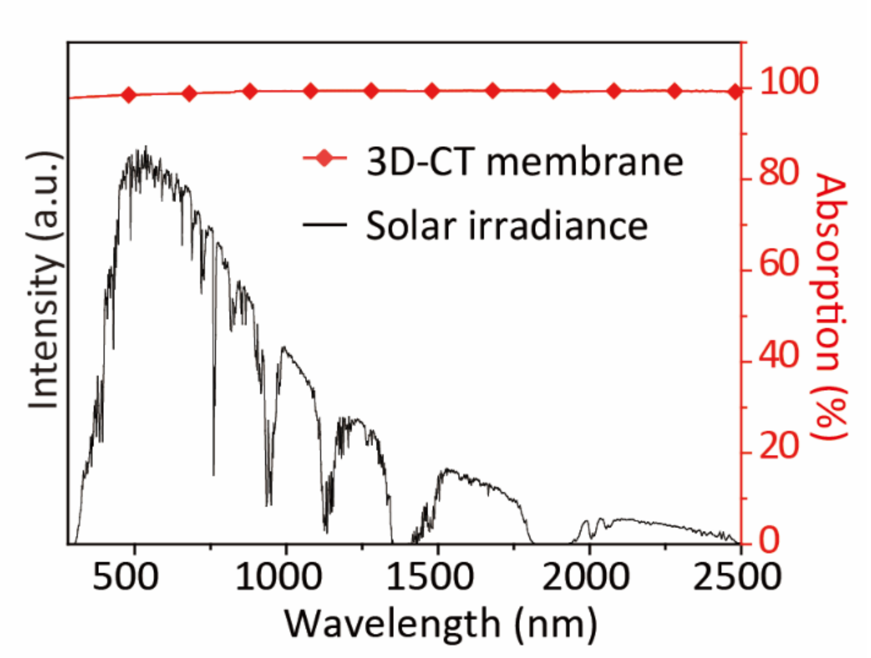

Figure S4. The solar intensity and absorption spectrum of the solar spectrum (280-2500 nm).

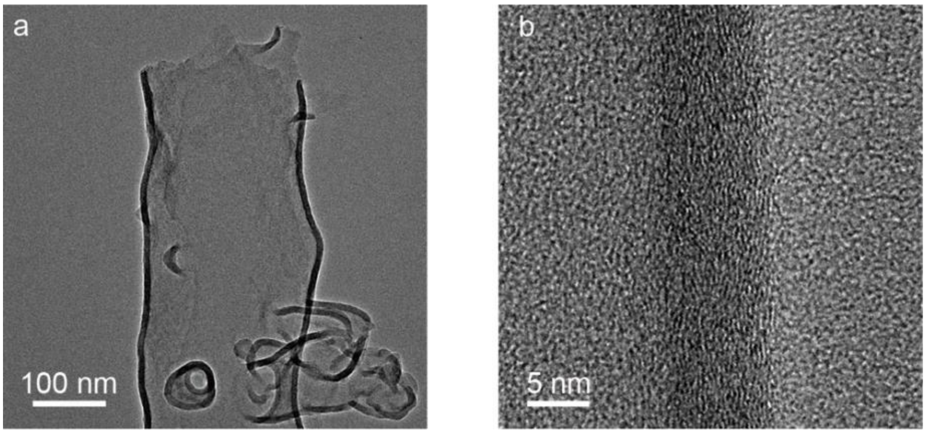

Figure S5. TEM characterization of the carbon tubes in the 3D-CT membrane, showing the uniform wall thickness.

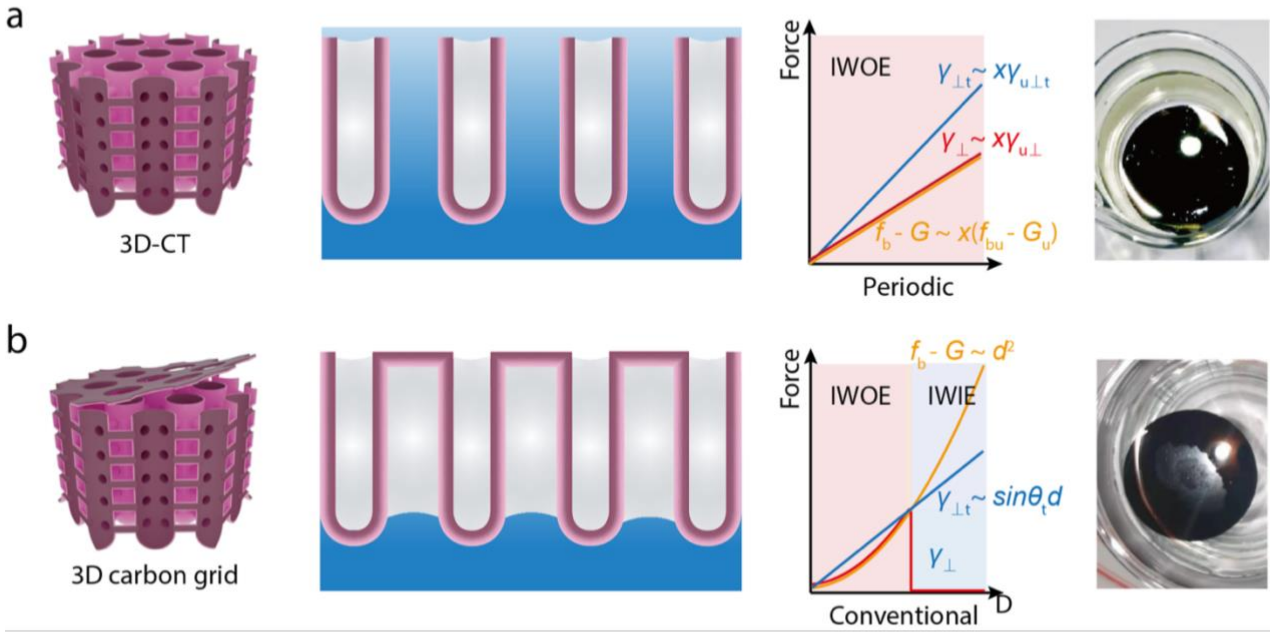

Figure S6. Working conditions of the 3D-CT (a) and 3D carbon grid (b) when they are immersed in the water. For 3D-CT, water can enter into and fill the space among CTs, showing scalable periodic structures under water for maintaining a suspending working state with an interfacial layer naturally existing on the top of the evaporator. However, for a 3D carbon grid, water cannot enter into the space aomong the CTs due to the carbon film blocking, making it fail to form the periodic structure under water. Therefore, a 3D carbon grid with large size floats on the water surface instead of suspending in water.

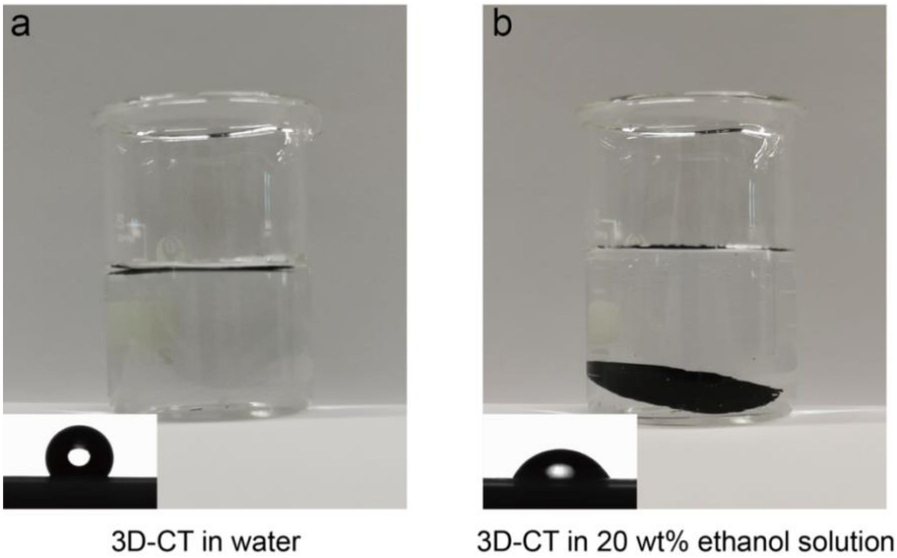

Figure S7. Working conditions of the 3D-CT in water (a) and 20 wt% ethanol solution (b). The 3D-CT suspends in the water with an interfacial layer naturally existing on the top of the evaporator. When placing it into 20 wt% ethanol solution, it sinks to the bottom.

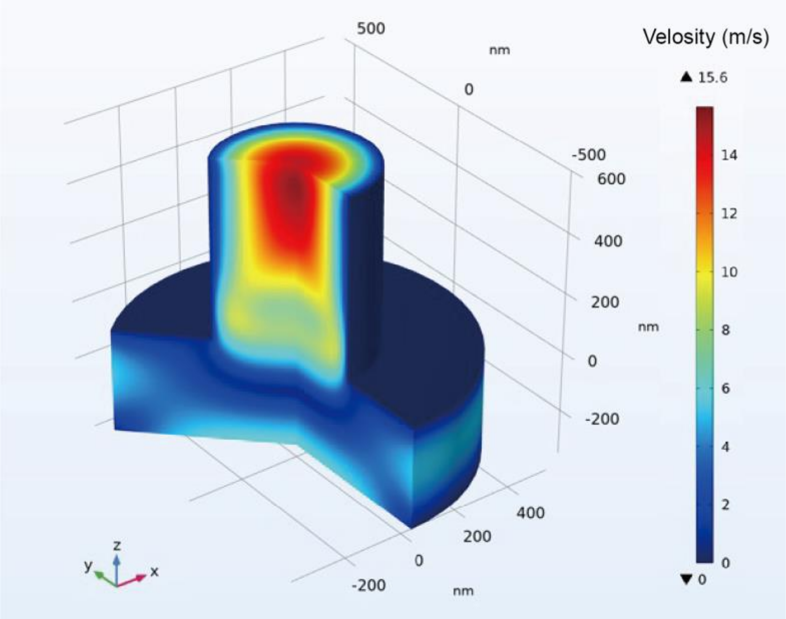

Figure S8. The simulation result of the water supply speed of the 3D-CT membrane with considerations of nanochannel size and viscosity of water.

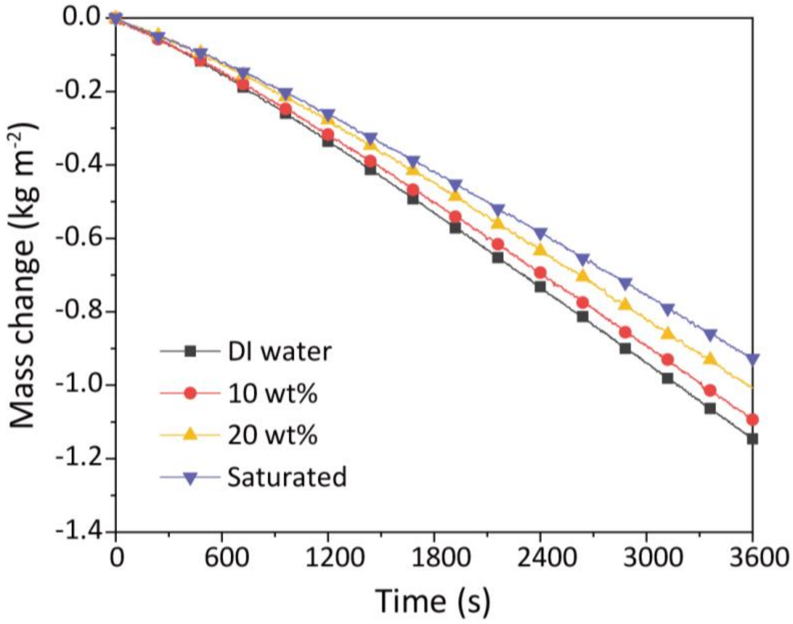

Figure S9. Mass changes of all the water resources (DI water, brine water with 10 wt% and 20 wt%, and saturated brine water, respectively) under 1 sun illumination.

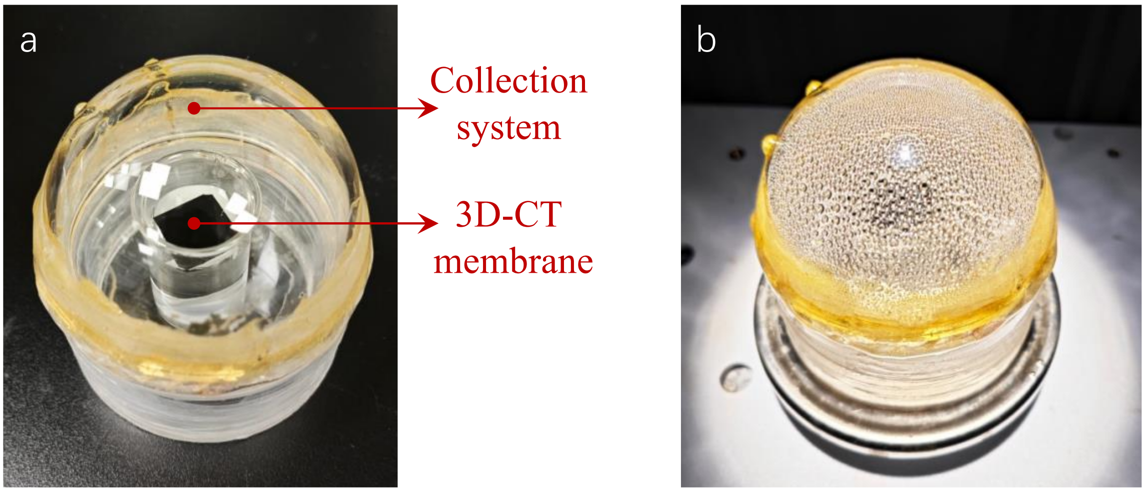

Figure S10. The complete evaporation-condensation-collection system for solar water purification. a, The photo and illustration of the system. b, The photo of the system after solar illumination.

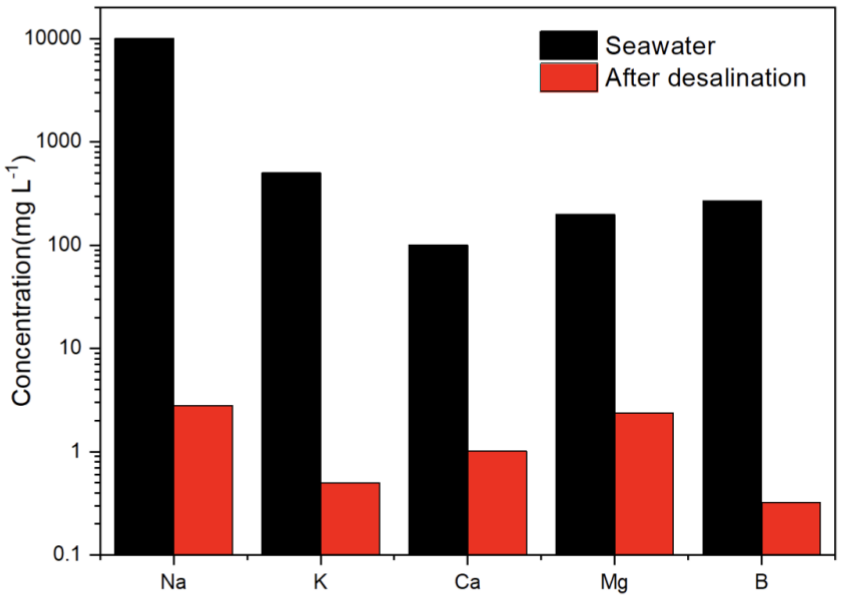

Figure S11. Concentrations of ions for seawater collected from the Bohai Sea and the water after solar desalination.

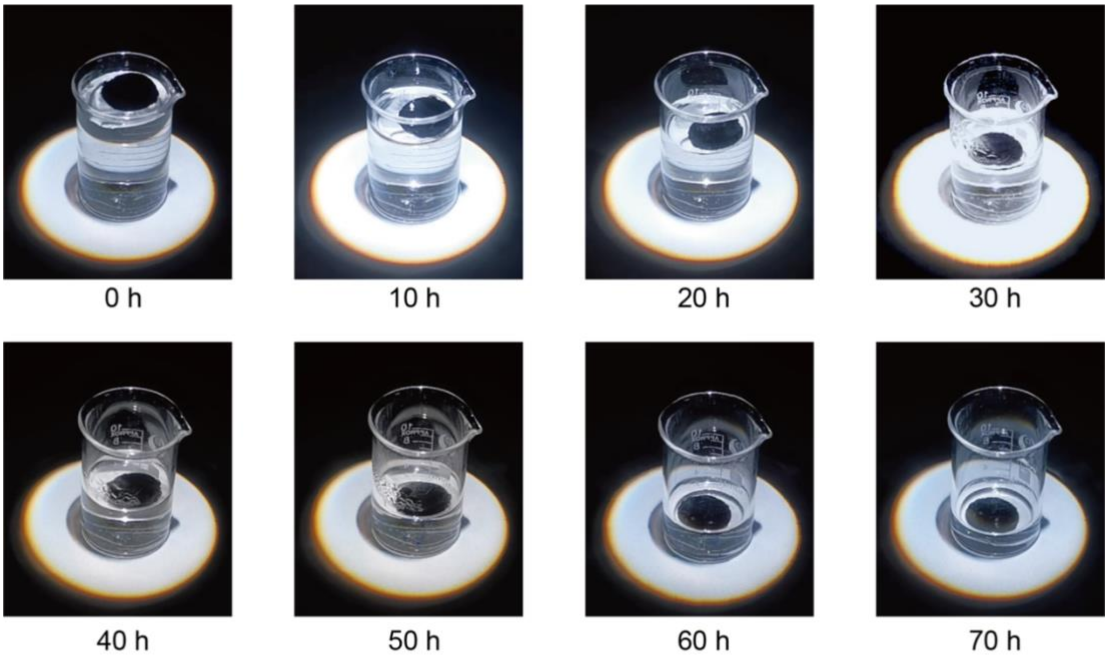

Figure S12. Photos of the 3D-CT when treating real seawater from the South China Sea.

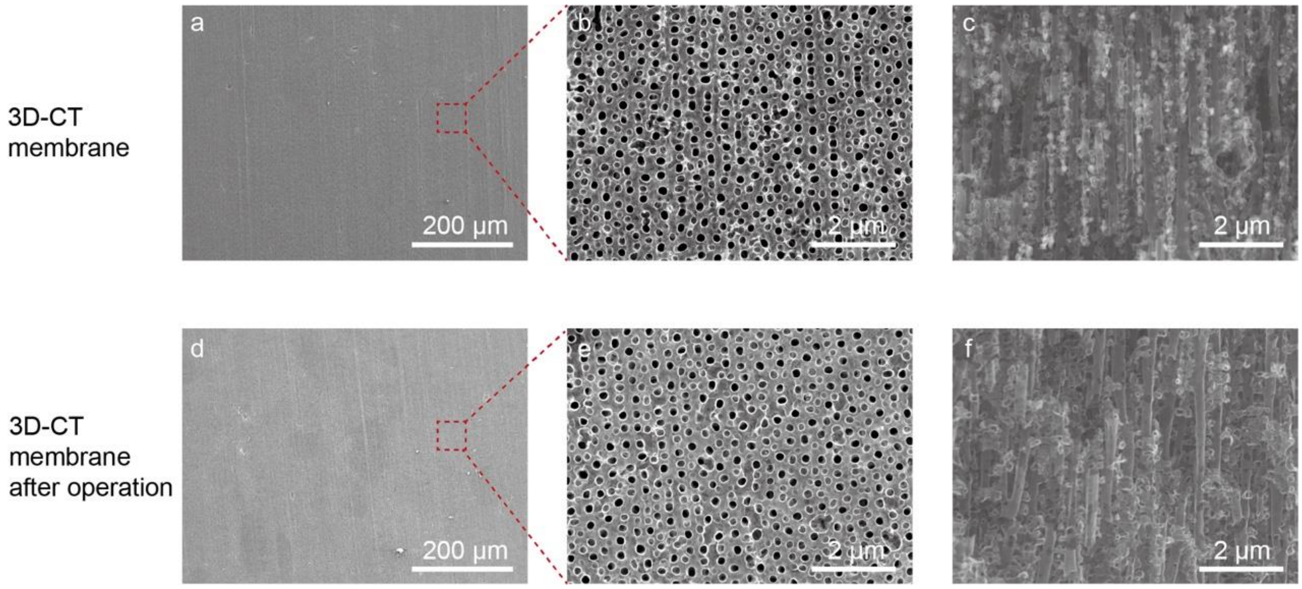

Figure S13. SEM images of 3D-CT membranes before and after 3 days of solar treatment of seawater. a and b, top view of the 3D-CT membrane. c, cross-sectional view of the 3D-CT membrane. d and e, top view of the 3D-CT membrane after solar treatment of sea water. f, cross-sectional view of the 3D-CT membrane after solar treatment of sea water.

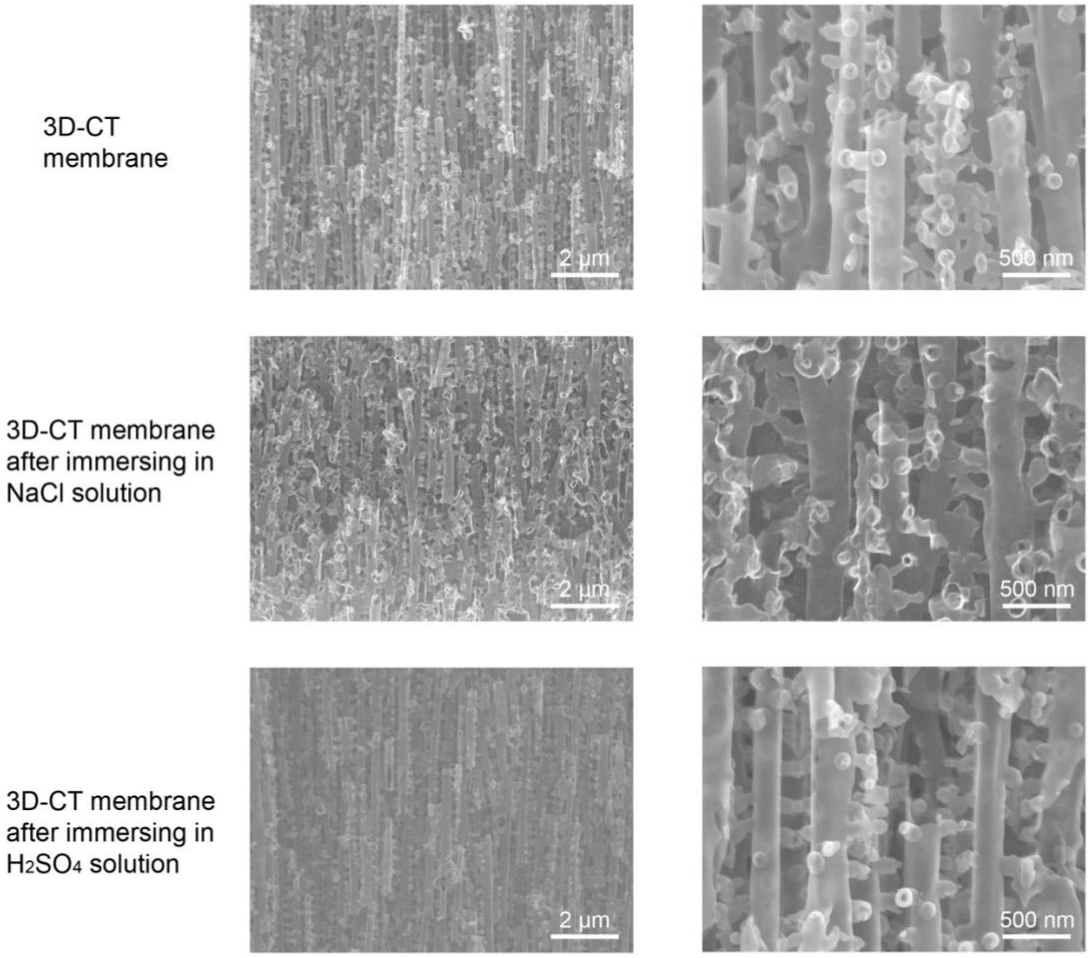

Figure S14. SEM images of the 3D-CT membranes before (a, b) and after being immersed in NaCl solution (c, d) and H_2SO_4 solution (e, f).

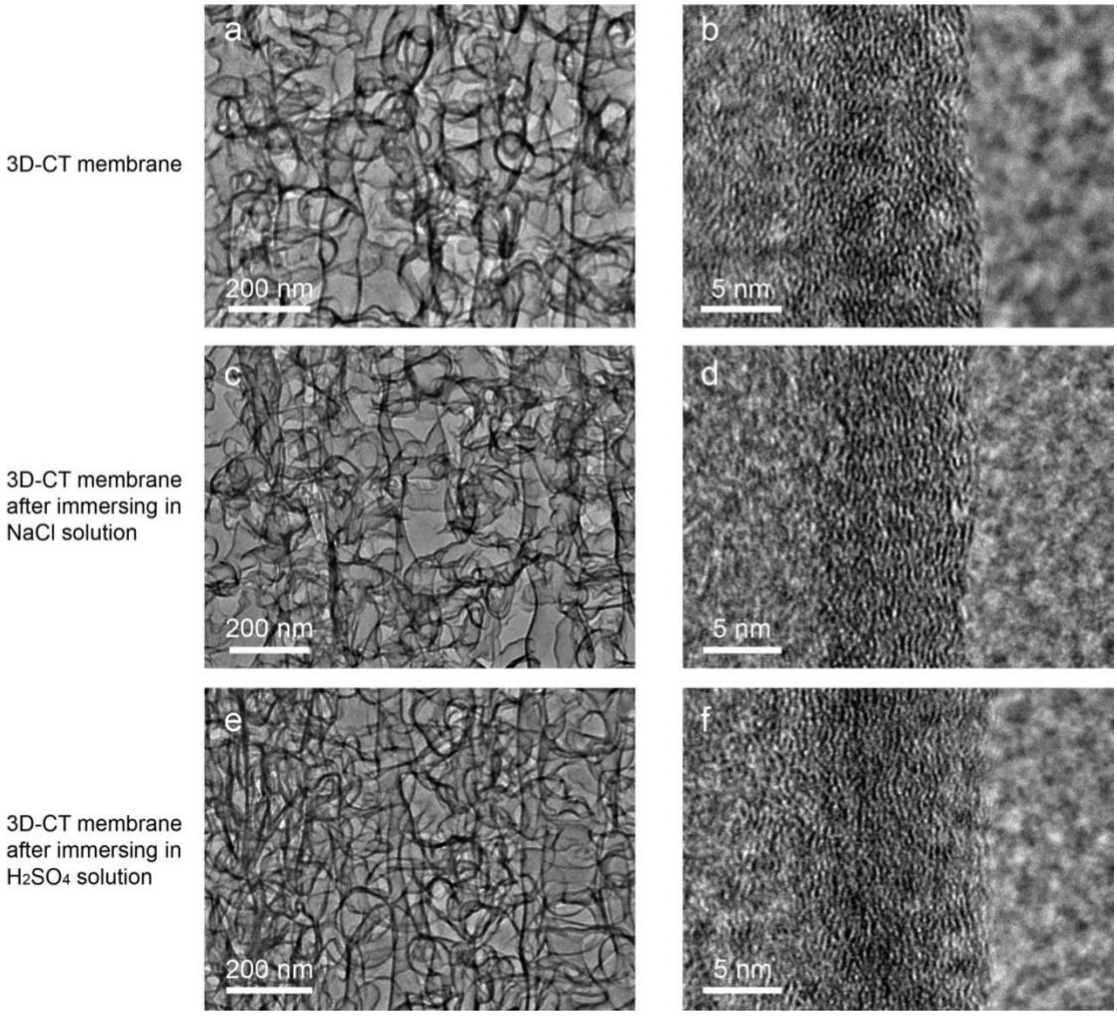

Figure S15. TEM images of the 3D-CT membranes before (a,b) and after being immersed in NaCl solution (c,d) and H_2SO_4 solution (e,f).

Supplementary Notes

Supplementary Note 1. Structural defect tolerance and robustness analysis

We have performed analysis to evaluate how structural heterogeneities, defects, or partial wetting of the internal tube walls could affect the predicted equilibrium. Local structure heterogeneities and defects (e.g., broken walls, blocked tubes), as well as partial wetting of internal tube walls, may alter the buoyancy/gravity balance of individual tubes. Nevertheless, the periodic and interconnected architecture of the 3D-CT membrane enables it to behave as a collective system. Even if a small fraction of tubes loses air entrapment (due to wall defects or partial wetting of internal tube walls), the resultant loss in local buoyancy can be compensated by the upward force provided by surrounding intact tubes.

To quantify the tolerance of the membrane to such imperfections, we introduce a parameter p, defined as the fraction of heterogeneities/defects/partial wetting of the vertical CTs, whose inner cavities become water filled (i.e., lose buoyancy). The condition for the membrane to remain in the IWOE state is: $G_u < f_{bu}(1-p)$. Substituting the expressions for gravity and buoyancy per tube gives: $\rho_c \pi D_1 \Delta D\, hg < \frac{\pi}{4} D_1^2 h \rho_w g\,(1-p)$. If the inequality is reversed, the membrane would sink and lose the IWOE working state. Using the same ranges of geometric and material parameters as demonstrated in manuscript ($D_1 \approx$ 200–300 nm; $\Delta D \approx$ 5–10 nm; $\rho_c \approx$1.8–2.3 g cm^{-3}), a simple estimate indicates that sinking would require a high fraction of defective or wetted tubes—approximately 54%–88% (depending on the actual values of D_1, ΔD, and ρ_c). In other words, the membrane remains IWOE working state as long as p (extent of heterogeneities/defects/partial wetting) less than about 54% of the vertical tubes. This demonstrates that the system possesses considerable robustness against localized imperfections.

Supplementary Note 2. The stable working state of the interfacial water layer on top

The working state of the membrane is governed by the buoyancy, gravity, and surface tension of the membrane underwater. There are all the inherent properties of the membrane, which are determined by the structures and wetting properties of the 3D-CT membrane and will not be affected by the environment.

As shown in fig. S16, the 3D-CT membrane maintains the suspended working state with an interfacial water layer on top, even at the wind speed of ~2.5 m s^{-1}. The experiment in the wind is also recorded by video, which has been added as Supplementary Video 2 in the Supporting Materials.

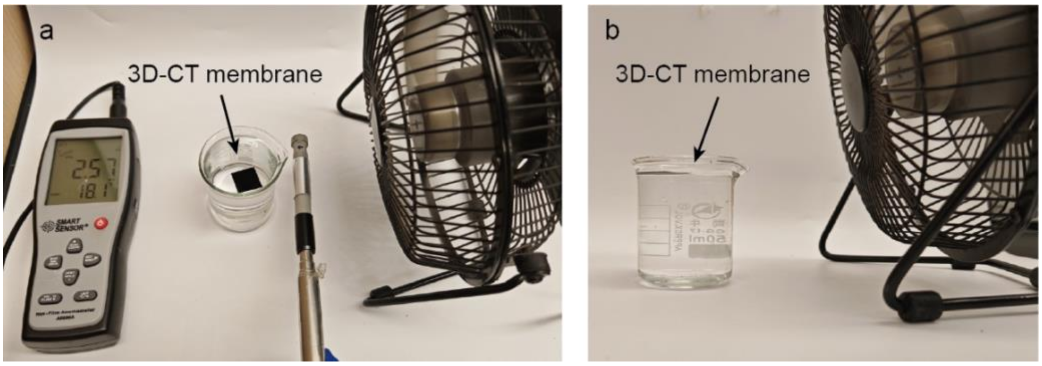

Figure S16. The top view (a) and side view (b) illustrate that the 3D-CT membrane maintains the suspended working state with an interfacial water layer on top in the wind.

We also shake the water to simulate the wave. The 3D-CT membrane also demonstrates a stable suspended working state, as shown in fig. S17. The corresponding video of the experiment is also added as Supplementary Video 3 in Supporting Materials.

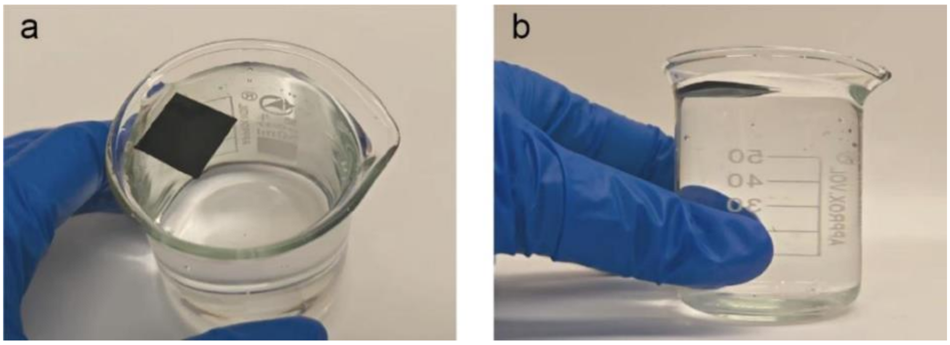

Figure S17. The top view (a) and side view (b) demonstrate that the 3D-CT membrane maintains the suspended working state with an interfacial water layer on top in the water wave, which is generated by shaking.

Temperature variation also shows little effect on the mechanical state of the 3D-CT membrane. It observed that the 3D-CT membrane maintains a suspended working state with an interfacial water layer on top in the water at room temperature and at 75ºC.

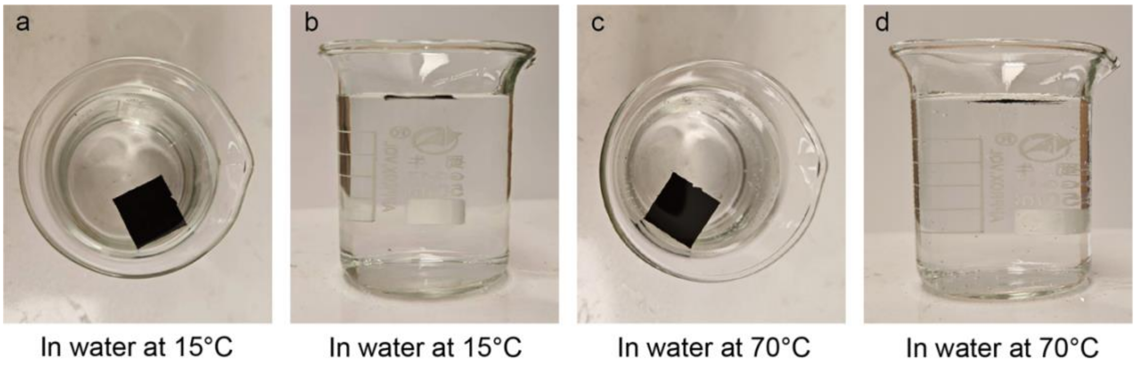

Figure S18. The top view (a, c) and side view (b, d) demonstrate that the 3D-CT membrane maintains the suspended working state with an interfacial water layer on top in the water at room temperature (15ºC) and at 75ºC.

Supplementary Note 3. Heat loss analysis

1. Conductive heat Loss

In the IWOE design, the 3D-CT membrane is suspended just below the air–water interface, with a thin interfacial water layer above it. Since the membrane is in direct contact with water both above (interfacial layer) and below (bulk water), conductive heat transfer occurs primarily through these water layers. The heat conducted upward into the interfacial water layer contributes to evaporation, while the heat conducted downward into the bulk water constitutes conductive heat loss. This downward heat flux can be estimated using Fourier's law, $q=-k\nabla T$. We measured the temperature of the bulk water at two vertical positions. A temperature difference of 3.2°C was observed over a height difference of 2 cm, corresponding to a conductive heat flux of approximately 92.8 W m^{-2}, assuming thermal conductivity of water of 0.58 $W \cdot m^{-1} \cdot K^{-1}$. This conductive heat loss accounts for about 9% of the incident solar flux.

2. Radiative heat Loss

The membrane is submerged beneath a thin water layer. It transfers heat to the above interfacial water layer via heat conduction and radiation, and subsequently from the water surface to the ambient via radiation. The emissivity of both the membrane and water is estimated as $\varepsilon \approx 0.95$, and the temperature of the membrane surface (almost equivalent to the water surface temperature) is characterized as $T_m \approx$ 40 °C. The ambient temperature is characterized as $T_a \approx 28$ °C According to the Stefan–Boltzmann law, $q = \varepsilon\, \sigma\, (T_m^4 - T_a^4)$, the radiative heat flux is calculated to be 74.83 W m^{-2}. This radiative heat loss accounts for approximately 7% of the incident solar flux.

3. Convective heat Loss

Convective heat loss was calculated using Newton's law of cooling, $q = h\Delta T$, with an estimated heat transfer coefficient of $h = 5 \text{ W m}^{-2}\text{ K}^{-1}$. The resulting convective heat flux is 60 W m^{-2}, accounting for approximately 6% of the incident solar flux.

Supplementary Note 4. Salt flux analysis

In our designed 3D-CT membrane, the solar brine treatment process operates in two periods with different self-cleaning strategies: salting-free and salting-out. When treating brine of moderate concentration (e.g., 10 wt%), ion diffusion across the interfacial layer is sufficiently rapid to redistribute ions away from the evaporation front, meaning the ion diffusion flux keeps pace with the evaporation-driven ion accumulation. This prevents local supersaturation from reaching the critical nucleation point, thereby avoiding salt precipitation entirely (namely salting-free process). When treating brine near saturation, ion diffusion becomes insufficient relative to the rapid concentration increase driven by evaporation. This imbalance leads to localized supersaturation and triggers the salt precipitation (salting-out) process. The grown salt crystals are then autonomously removed via the membrane's tilting mechanism, enabling self-cleaning.

To quantitatively compare ion diffusion and evaporation-driven concentration, we performed COMSOL simulations. The 3D-CT membrane was modeled as being placed on an infinite brine reservoir with varying initial salinities. Figure S19a shows the steady-state evaporation-driven upward salt flux and the diffusion-driven downward salt flux as functions of the brine concentration. For most salinities, the two fluxes balance, consistent with the salting-free behavior. However, when the brine approaches saturation, the concentration gradient between the top and bottom become too weak to sustain a sufficient diffusive back-flux, leading to net salt accumulation and precipitation. Here, we examined two representative cases in detail.

First, we take the solar treatment of 10 wt% brine as an example of the salting-free process, analyzing the competition between evaporation-driven ion accumulation and back-diffusion via COMSOL simulation. Under 1-sun illumination, the evaporation rate of the 3D-CT membrane is ~1.21 kg m^{-2} h^{-1}. This water loss translates into a local concentration increase at the evaporation front. Ion transport (Na^+, Cl^-) follows Fick's law with a diffusion coefficient of ~1×10^{-9} m^2 s^{-1}. The simulation reaches steady state within seconds, where the downward diffusive flux exactly compensates the upward advective flux, maintaining a salt-free evaporation interface (fig. S19b, c).

Besides, we take solar treatment of saturated brine (26.5%) as an example of the salting-out process. The evaporation rate decreases slightly to ~1.04 kg m^{-2} h^{-1}. However, because the brine is already near saturation, the concentration gradient that drives back-diffusion is greatly diminished. As a result, the diffusive flux can no longer offset the evaporation-driven ion accumulation, leading to rapid supersaturation and salt precipitation at the top surface (fig. S19d, e). These precipitated crystals then initiate the self-cleaning tilt.

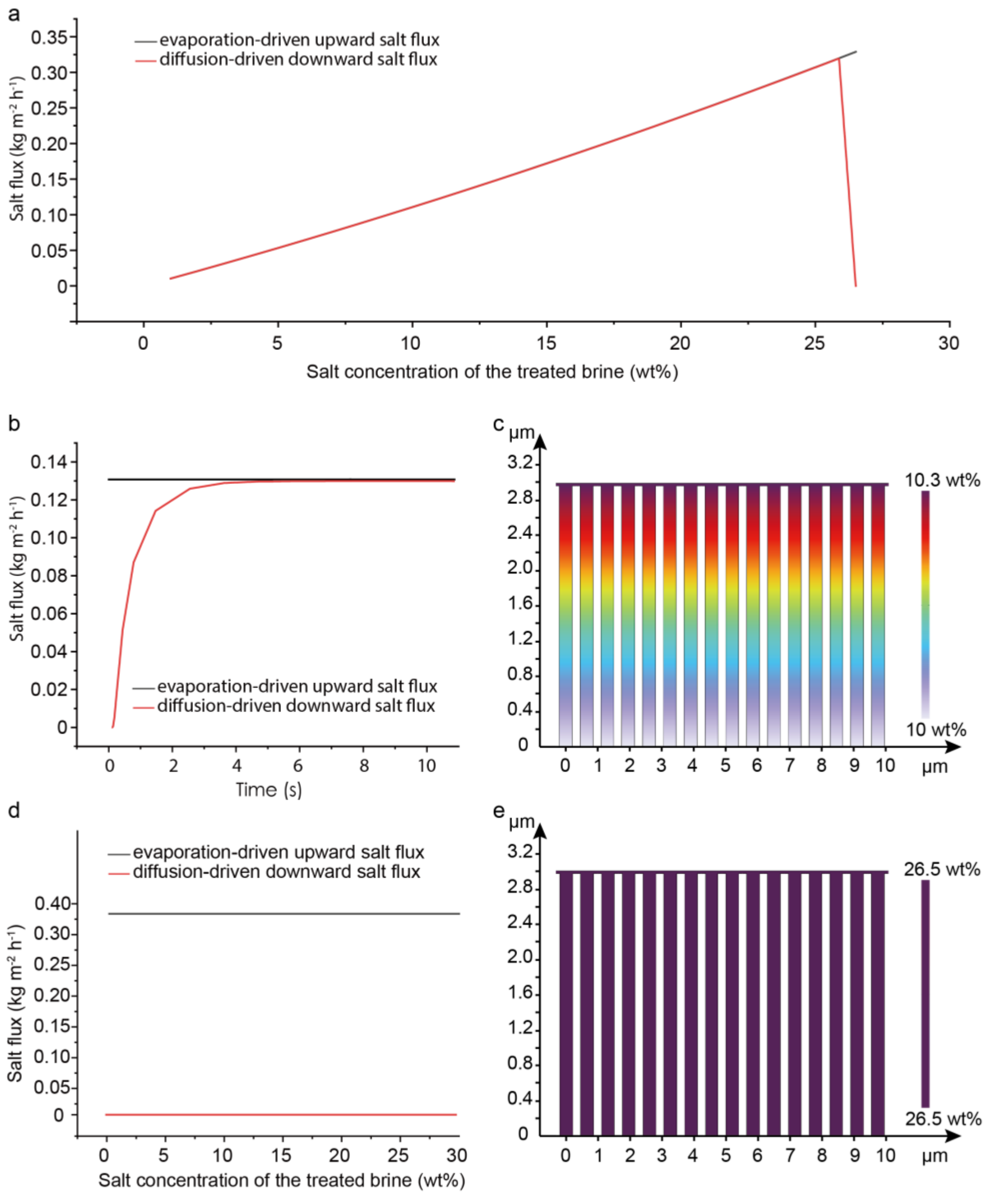

Figure S19. a, Salt flux in the interfacial water layer as a function of feed salinity. b, and c, Evaporation-driven upward salt flux and diffusion-driven downward salt flux (b), and the corresponding steady-state concentration profile (c) for 10 wt% brine. d, and e, Respective salt fluxes (d) and concentration profile (e) for saturated brine (~26.5 wt%).

Supplementary Note 5. Reproducibility of the self-cleaning process

Once salt forms, the resulting torque is uniquely determined by the position of its center of mass relative to the membrane edge. For clarity, we consider a membrane of 20 mm in diameter for our analysis. Through mechanical modeling, we analyze the tilt-onset time t_1 and the salt-gliding time t_2 as functions of the lever arm distance r (the horizontal distance from the crystal's center to the pivot edge, OC, as shown in fig. S20a). As illustrated in fig. S20b, for nearly all off-center crystal positions (i.e., $r > 10$ mm), both t_1 and t_2 remain finite. This confirms that the membrane will inevitably tilt and eventually cause the crystal to glide off, regardless of where the salt initially precipitates.

A perfectly centered salt deposit (theoretically $r \to 10$ mm) would produce negligible torque and might not trigger tilting, but such symmetric configuration is statistically very rare under realistic evaporation. In practice, crystal growth occurs within thin interfacial water layer and naturally results in an off-center mass distribution. The self-cleaning outcome is therefore robust and reproducible, as consistently observed in our repeated experiments (Fig. 5e and Supplementary Video 1).

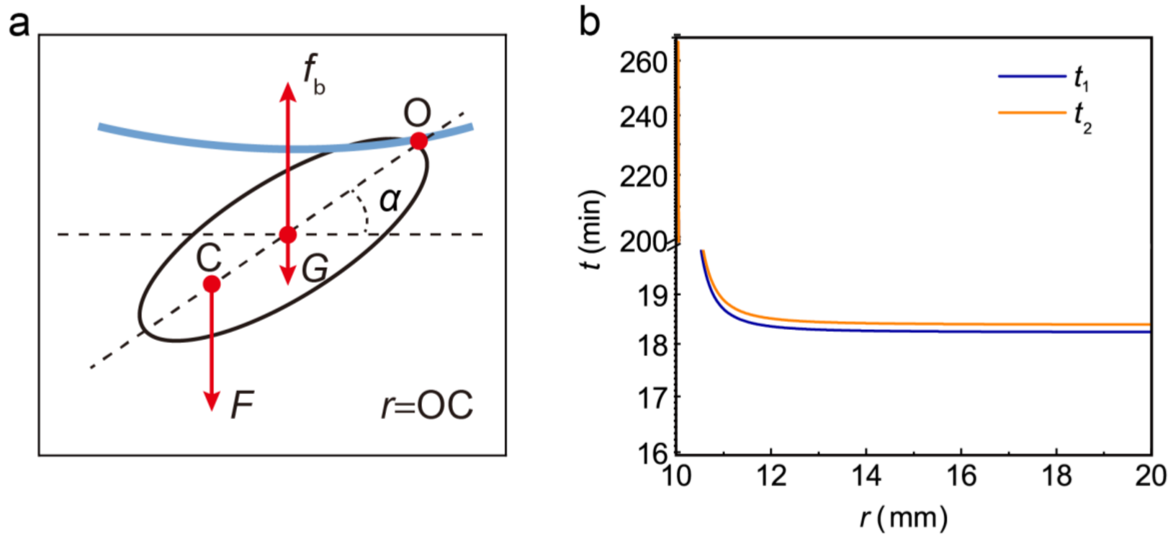

Figure S20. a, Mechanical analysis of the self-cleaning process. b, Predicted tilt onset and salt-gliding times versus precipitation location. The lever arm r is the distance from the precipitation mass center C to the edge pivot O. Solid curves show the predicted t_1 and t_2 trends over $r = 10 - 20$ mm for a 20-mm-diameter membrane, where t_1 denotes the onset of rotation and t_2 denotes the onset of salt gliding/removal.

Supplementary Note 6. Alternative material systems for scalable IWOE implementation

In consideration of the scalability, materials with low cost and commercial setup are preferred. Inspired by the periodic 3D CT membrane which serves as a demonstration, other materials with a similar mechanical balance under water can be designed and fabricated for further scalable application. These materials also require elaborate design to realize the mechanical balance of buoyancy, gravity, and surface tension.

Here, we proposed a PPy-PDA-coated EVA membrane with periodic holes for scalable solar water treatment. Ethylene Vinyl Acetate (EVA) is a type of commercial material for fabricating membranes with density below 1 g cm^{-3}, which is expected to meet the requirements through structure design and surface modification. Polypyrrole (PPy) coatings are used for enhancing solar absorption (fig. S21) and wetting property (fig. S22), while polydopamine (PDA) serves as an adhesive binder between PPy and EVA to enhance the adhesion of PPy on the EVA surface. It can be seen that the normalized light absorption of the PPy-PDA-coated EVA membrane with water film above is tested to be over 97% (weighed by solar spectrum, fig. S21).

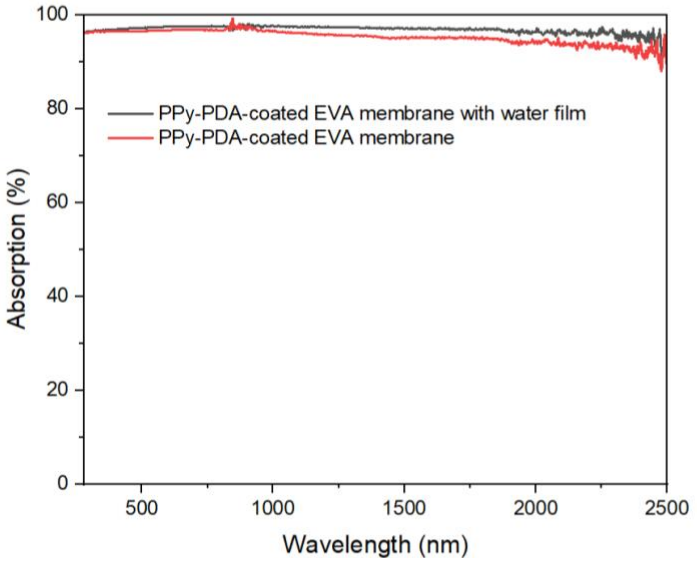

Figure S21. The absorption spectra of the PPy-PDA-coated EVA membrane and the membrane with water film.

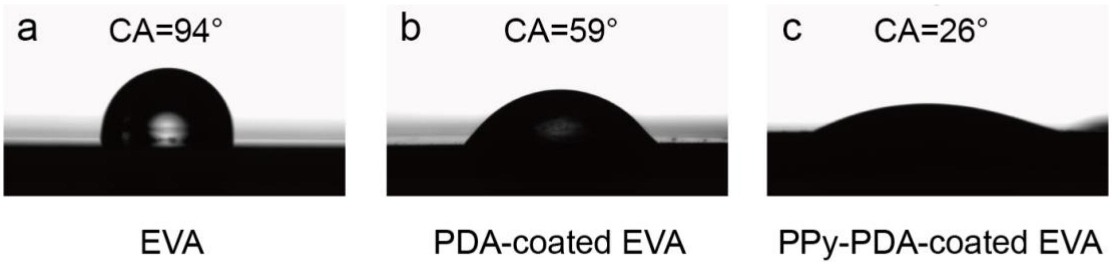

Figure S22. The contacting angle of EVA membrane (a), PDA-coated EVA membrane (b), and PPy-PDA-coated EVA membrane.

The PPy-PDA-coated EVA membrane with hole diameter of 1 mm (R_p= 0.5 mm), periodicity of 1 cm (D = 5 mm), and thickness of 0.4 mm (d = 0.4 mm) is chosen for meeting the mechanical balance for suspended working state. As shown in fig. S23, the membrane can successfully suspend in water with interfacial water layer on top of the evaporator. The EVA-based membrane is industrially mature and producible, and the ppy decoration is a solution synthesis method, which is easy to scale up. Thus, PPy-PDA-coated EVA membrane with periodic holes possesses excellent scalable capabilities.

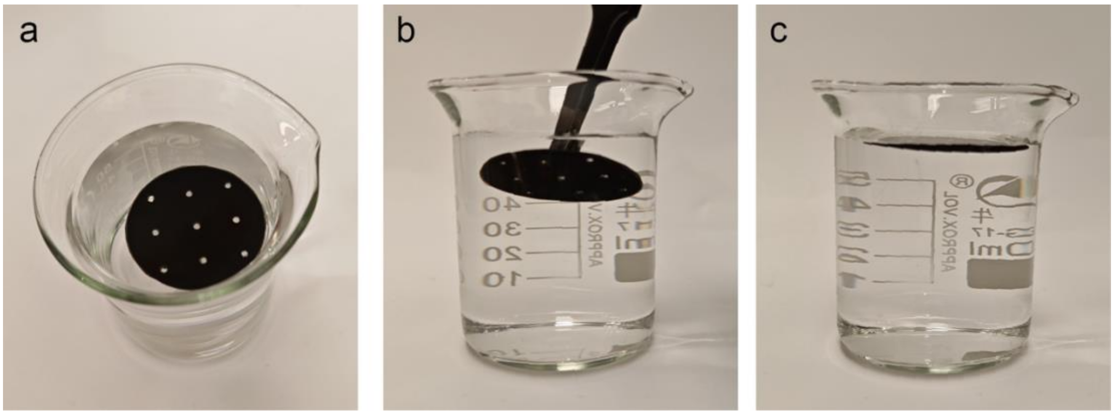

Figure S23. The PPy-PDA-coated EVA membrane with periodic holes (a). Optical photos show that the membrane exhibits suspended working state under water (b, c). It won't sink even after being pressed into the deep water (b), and there exits an interfacial water layer on the top of the PPy-PDA-coated EVA membrane (c).
